# Multiple sclerosis in a 4-year-old boy: a case report and literature review

**DOI:** 10.3389/fneur.2024.1359938

**Published:** 2024-03-22

**Authors:** Ula Arkar, Tina Vipotnik Vesnaver, Damjan Osredkar, Mirjana Perković Benedik, Neli Bizjak

**Affiliations:** ^1^Department of Child, Adolescent and Developmental Neurology, University Children’s Hospital, University Medical Centre Ljubljana, Ljubljana, Slovenia; ^2^Department of Radiology, University Medical Centre Ljubljana, Ljubljana, Slovenia; ^3^Faculty of Medicine, Center for Developmental Neuroscience, University of Ljubljana, Ljubljana, Slovenia

**Keywords:** multiple sclerosis, pediatric onset multiple sclerosis, very early onset multiple sclerosis, disease modifying therapies, rituximab

## Abstract

Pediatric onset multiple sclerosis (POMS) in the very young is a very rare entity and presents a difficult diagnostic challenge due to overlapping signs and symptoms with other diseases. We present a 4-year-old boy who initially presented with right-sided hemiparesis and demyelinating lesions on MRI. Follow-up MRI examinations 3 and 6 months later revealed new demyelinating lesions. Ten months after initial presentation, he presented with right-sided hemiparesis, central facial nerve palsy on the right side and new demyelinating lesions on MRI. Two clinical events and new MRI lesions on follow-up MRIs confirmed the diagnosis of POMS. He was treated with rituximab and experienced no further relapses or radiological progression during the follow-up period.

## Methods

We conducted a comprehensive review of the relevant scientific literature. The search was conducted using PubMed, Scopus, and Google Scholar databases. The literature search was conducted in two steps. First, a naive search was conducted using Boolean search queries. No filters or other search restrictions were applied. The last search was conducted in March 2023. The final selection of the top-k studies was done manually by the authors (UA and NB). We included case reports, case series, original research articles and review articles.

## Literature review

Pediatric onset multiple sclerosis (POMS), defined as multiple sclerosis (MS) with onset in patients younger than 18 years, occurs in 3–10% of all MS patients (1). The incidence varies by country and is estimated to range from 0.57 to 2.85/100000 children (2–5). Onset before the age of 10 is extremely rare (6–9) with an incidence of 0.2–0.7% (10).

### Clinical presentation

Unlike older children and adults, prepubertal children are more likely to present with brainstem involvement (1, 8) as well as polyfocal deficits and encephalopathy (11), making it difficult to distinguish MS from acute disseminated encephalomyelitis (ADEM), which is much more common in children younger than 10 years (10, 12). Huppke et al. compared 47 prepubertal and 41 postpubertal MS patients. The prepubertal patients were more likely to have a polysymptomatic severe first episode with motor and brainstem involvement, sphincter dysfunction, and cognitive disturbances, whereas the postpubertal patients predominantly presented with optic neuritis and sensory symptoms. They found no gender prevalence in prepubertal children (8). Öztürk et al. compared patients with preschool and school age onset (30 children). They observed a higher rate of motor symptoms and more attacks in the first year in preschool children (13).

### Radiologic and laboratory findings

Prepubertal patients also have unique CSF profiles and imaging findings (14). Chabas et al. found specific CSF findings with neutrophilic pleocytosis, higher proportion of monocytes, and absence of IgG synthesis in younger children with POMS (15). They found that changes in CSF immunoglobulins appeared more frequently with later relapses in children with disease onset under 10 years of age, whereas oligoclonal bands tended to become positive with increasing disease duration (15).

Prepubertal cases also have unique radiologic findings. The T2-bright foci on initial brain MRI tend to be ill defined and not ovoid, the deep gray matter is more frequently affected, and the number and size of T2-bright foci decrease dramatically on follow-up scans compared with teenagers (16).

### Diagnostic criteria

For the diagnosis of MS, the 2017 revised McDonald criteria (17) are currently used. The criteria are most applicable to patients older than 10 years. In younger patients, because of the far more common occurrence of ADEM, the criteria cannot be applied at the time of the first demyelinating attack and a second relapse characteristic of MS is necessary to confirm the diagnosis (17).

### Early case series and case reports

There are only a few recent case series of children with very early onset MS. Some case series were published several years ago (6, 7). It is important to emphasize that these case series were published before testing for MOG and aquaporin-4 antibody titers became widely available and recognized in clinical practice. Ruggieri et al. published an article in 1999 describing a cohort of 49 children with POMS under 6 years of age. In their cohort, 63% of the children experienced their second relapse in less than 1 year, and during the follow-up period (mean duration 6.8 years), only 64% had complete recovery (6). Individual case reports of very young patients with POMS who were treated only with methylprednisolone for relapses and had a poor neurological outcome have also been published (18). Although these studies describe well the natural course of POMS with very early onset, they do not tell us much about the prognosis of children diagnosed and treated today given the numerous new therapeutic options.

Many case reports also emphasize that diagnosis in patients with very early onset is often delayed. Sivaraman et al. described a patient with onset of MS at a very young age of 2 years and 1 month (10). She presented with acute onset of ataxia without encephalopathy. Her brain MRI revealed extensive white matter lesions that were disseminated in space, whereas her CFS was normal. She was misdiagnosed as having ADEM. She experienced two further relapses, and only after she was evaluated at another hospital for a second opinion, the diagnosis of POMS was established. Brain MRI revealed new areas of demyelination at each presentation, while oligoclonal bands remained negative. Treatment was not reported (10).

### Treatment

POMS also presents a treatment challenge (14) because of the lack of safety and efficacy data for disease-modifying therapies (DMTs) in children (19). In recent years, the treatment approach with early high-efficacy therapy (HEET strategy) has become more widely accepted in POMS than escalation regimen (19, 20), providing better disease activity control (19). For prepubertal patients, MyGinley et al. recommend the use of rituximab as first-line therapy, with maximization of dose and shortened treatment intervals for breakthrough disease activity (14). Treatment of POMS with rituximab is off-label (14, 21). In addition, the use of anti-B-cell therapies requires long-term follow-up to minimize the risk of serious adverse events, particularly the risk of immunoglobulin deficiency, malignancies, and infections (14). In children, some studies also suggest an increased risk of hypogammaglobulinemia with rituximab therapy (22, 23).

Multiple sclerosis in children under 10 years of age is extremely rare, and diagnosis is often delayed. Although there are some treatment recommendations for prepubertal children, treatment decisions are still largely dependent on local clinical practice. We present the case of a four-year-old boy with MS who was treated with rituximab and experienced no further relapse during the follow-up period.

## Case report

**Figure 1:**
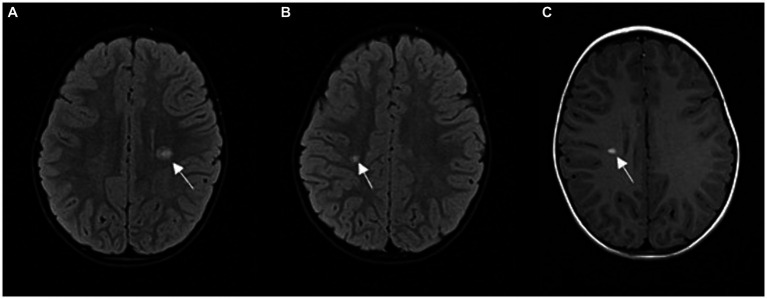
**(A,B)** FLAIR sequence in axial plane at two different levels; hyperintense demyelinating lesions **(A)** periventricular, above the left lateral ventricle (arrow), **(B)** in the deep white matter of the right centrum semiovale (arrow); **(C)** T1 contrast enhancement sequence in axial plane; homogeneous contrast enhancement of an active lesion (arrow).

The patient presented to our pediatric neurology department at the age of 4 years and 6 months after experiencing 1 week of progressive right-sided hemiparesis. The parents reported that the boy had been clumsy for a week before admission. On the morning of the day of admission, he could no longer grasp objects with his right hand, he could no longer lift things, and they noticed an increasing limp in his right leg. He is the third child of non-consanguineous parents. His mother had clinically isolated syndrome (CIS) 10 years earlier – she experienced one episode of optic neuritis with demyelinating lesions seen on head MRI that did not fulfill the criteria for dissemination in time and space. The boy’s perinatal history and early development were unremarkable. Neurologic examination revealed right-sided hemiparesis, symmetrically challenged tendon reflexes. Walking was limping. Mental status, cranial nerve assessment, and coordination were normal. He reported no sensory deficits. EDSS was 3.0. MRI of the brain revealed three demyelinating lesions in the corona radiata on the right side, in the centrum semiovale on the left side, and a parietal subcortical lesion on the left side. All of them displayed gadolinium enhancement (Figure 1). MRI of the spinal cord revealed one short demyelinating lesion in the lateral cervical medulla with longitudal length of 0.5 cm, without gadolinium enhancement. Laboratory studies showed CSF pleocytosis with lymphocyte predominance. Oligoclonal bands in the CSF and serum were negative, the method used was Isoelectric Focusing (IEF), followed by immunofixation. Serum laboratory tests were normal with low inflammatory markers, metabolic and rheumatologic tests were normal, and microbiological tests of serum and CSF were negative. Aquaporin-4-IgG and MOG-IgG antibody titers determined using cell-based assays were negative in CSF and blood serum. The ophthalmologic examination was normal. ADEM was suspected, although he did not meet the International Consensus criteria for ADEM (24). He was treated with high-dose intravenous methylprednisolone for 5 days, followed by an oral steroid taper for 4 weeks. He was also treated with intravenous immunoglobulins (IVIG). At discharge 12 days after presentation, he still had some reduced strength of the right hand, especially of the distal muscles, and poorer coordination and fine motor skills of the right hand. The gait was normal, and EDSS at discharge was 2.0. The patient was admitted to an intensive rehabilitation program. He continued to receive monthly IVIG applications. The initial management and treatment of the patient was at the discretion of a pediatrician not specialized in the treatment of pediatric demyelinating diseases.

Follow-up brain MRI 3 months later showed dissemination of the lesions in space. Six months after the right-sided hemiparesis, another MRI of the brain showed further dissemination of the lesions in space. By this time, he had already received 6 monthly IVIG applications, and the treatment was discontinued. At that time, the patient was referred to a pediatrician specialized in the treatment of pediatric demyelinating diseases.

**Figure 2:**
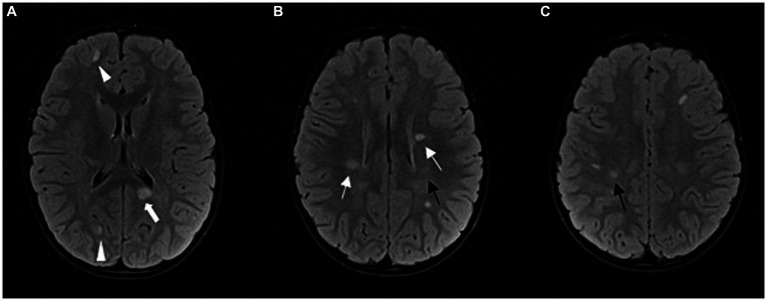
Follow-up MRI after 1 year: FLAIR sequence in axial plane at three different levels **(A–C)**; progression of the disease; multiple demyelinating lesions in typical periventricular (arrows) and juxtacortical locations (arrowheads). Among the periventricular lesions, we can observe a lesion in the splenium of the corpus callosum on the left side (thick arrow). Other new lesions can also be observed in the subcortical and deep white matter of the frontoparietal lobes. The lesions shown in Figure 1 have partially regressed (black arrows).

Ten months after his initial presentation, he presented with central facial nerve palsy on the right side and right-sided hemiparesis, EDSS was 2.0. A brain and spinal cord MRI revealed 5 new lesions, two of them displayed gadolinium enhancing (Figure 2). Laboratory studies showed mild CSF pleocytosis with decreased glucose in the CSF and normal protein concentration. Oligoclonal bands in the CSF and serum were negative, the method used was Isoelectric Focusing (IEF), followed by immunofixation. MOG-IgG and aquaporin-4-IgG antibody titers, determined using a cell-based assay in the CSF and serum, were negative. Serum laboratory tests were normal, and microbiological tests of the serum and CSF were negative. Metabolic and rheumatologic testing were normal. He was treated again with high-dose intravenous methylprednisolone for 3 days, followed by a complete clinical response, EDSS was 0. Monthly IVIG applications were resumed. The patient was referred to a clinical geneticist to exclude a possible genetic etiology of his condition. Whole exome sequencing (WES) followed by targeted gene panel analysis for neurodegenerative disorders yielded negative results.

**Figure 3:**
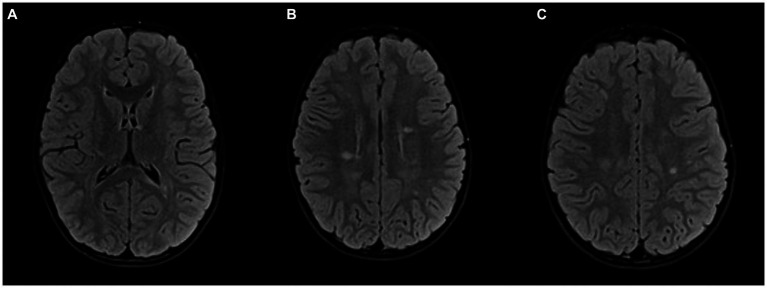
Follow-up MRI after 2 years: FLAIR sequence in axial plane at the same levels as in Figure 2 **(A–C)**; Partial regression of demyelinating lesions after initiation of therapy.

Seven months after the second clinical event, follow-up MRI of the brain and spinal cord showed multiple new demyelinating infratentorial lesions. Given that he had two clinical events and dissemination in time and space on several follow-up MRIs, he met the 2017 McDonald’s criteria. Eighteen months after the first clinical episode, after a thorough discussion with the patient’s parents, we decided to start rituximab therapy. Before starting rituximab, we performed several laboratory tests – complete blood count with differential, lymphocyte subpopulations, liver and kidney function tests, hepatitis B, C and HIV screening, immunoglobulin levels, tuberculosis tests, VZV serology. Once the safety of this drug for the patient was assured, treatment with rituximab was started, using 500 mg per meter squared of body surface (maximum dose of 1,000 mg per dose), dosed on days 1 and 15, and then every 6 months (14). A follow-up MRI 6 months after rituximab administration showed regression of the multiple demyelinating lesions and no new lesions (Figure 3).

**Figure 4:**
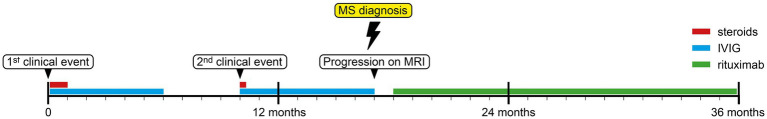
Case timeline.

The patient was invited for regular follow-up examinations every 6 months in our department, which consisted of a clinical examination, an MRI and an extended blood laboratory analysis (complete blood count with differential, lymphocyte subpopulations, liver and kidney function tests and electrolyte panel). No serious adverse events (SAEs) occurred during the follow-up period, but he suffered a local skin infection with *S. aureus*, which had to be treated with systemic antibiotics. At the last clinical examination, 36 months after initial presentation, he was clinically asymptomatic, his EDSS was 0 and his several follow-up MRIs showed no dissemination in space and time. He had already received three doses of rituximab and had not relapsed after starting this therapy (Figure 4).

Over the past 3 years, the patient and his parents have shown exceptional willingness to adhere to the treatment plan, expressing satisfaction with the medical care received, and gratitude for the support provided. National insurance fully covered all costs of medical treatments, ensuring that financial concerns did not hinder the patient’s care. Additionally, psychological support services were provided as part of the standard care. The boy has been able to live a normal life without neurological deficits and can actively participate in school and sports alongside peers. The boy’s parents expressed keen interest in sharing their story through this case report to raise awareness of POMS in the very young, underlining the importance of early diagnosis and comprehensive care in achieving favorable outcomes.

## Discussion

Pediatric onset multiple sclerosis (POMS) in the very young is a very rare entity and presents a difficult diagnostic challenge due to overlapping signs and symptoms with other diseases (2–5, 10, 25). Any delay in diagnosis can have a significant negative impact on the child’s neurologic outcome.

Our patient first presented at the age of 4 years with a typical monofocal nonencephalopathic clinical event (subacute hemiparesis). This was accompanied by MRI findings that met the 2017 Revised McDonald criteria for dissemination in space with more than one supratentorial lesion and one infratentorial lesion, as well as with criteria for dissemination in time, as all supratentorial lesions displayed gadolinium enhancement. Lumbar puncture showed CSF pleocytosis with negative oligoclonal bands, which is a common finding in prepubertal children (15).

ADEM was initially suspected in our patient because it is a more common demyelinating disorder in this age group and a common misdiagnosis in MS patients with very early-onset (10, 12, 26). Certain MRI findings can help differentiate pediatric patients with MS from those with ADEM at their first clinical event (27). The presence of T1 hypointense lesions, T2 periventricular lesions, and brainstem lesions have been shown to be very specific and sensitive predictors of MS diagnosis in pediatric patients with acute demyelinating events (27, 28).

His subsequent MRIs showed new demyelinating lesions. Ten months after his initial presentation, he presented with his second clinical event and new demyelinating lesions, which confirmed the diagnosis of multiple sclerosis (17).

Distinguishing between POMS and other demyelinating diseases such as MOGAD in very young children is pivotal for implementing appropriate therapeutic strategies and predicting long-term outcomes. Recent studies underscore the importance of testing for MOG-IgG and aquaporin-4-IgG in young pediatric patients presenting with a demyelinating event (25, 29). Research by Fadda et al. found that MOG-IgG is detectable even in children who meet the 2017 McDonald diagnostic criteria for MS, highlighting the need to test for the presence of MOG-IgG at the first clinical event to avoid misdiagnosis (25). Furthermore, a study by Yilmaz et al. found a decrease in POMS in children younger than 12 years, likely attributable to the recognition of patients with antibody-mediated disease (29). This evolving understanding emphasizes the importance of incorporating MOG-IgG and aquaporin-4-IgG testing into the diagnostic evaluation of pediatric demyelinating diseases.

Currently, only two MS DMTs (fingolimod and teriflunomide) have been tested in large phase III trials and approved by regulatory agencies for use in POMS (30). There is growing evidence that patients with POMS need early treatment with highly effective disease-modifying therapy (DMT) to prevent significant long-term disability (31, 32). However, an unanswered question is the use of newer DMTs in patients younger than 10 years of age. In recent years, only a few case reports and case series have been published on children with prepubertal MS (8, 13), making it difficult to decide on the most appropriate treatment in this age group. Although there are some treatment recommendations for prepubertal children (14), treatment decisions still depend largely on local clinical practice. Our decision to use rituximab was guided by the recommendations in the review by McGinley and Rossman (14). Additionally, our decision was influenced by the limited experience with other DMTs in prepubertal children, whereas rituximab is a well-established and time-tested therapy that is frequently used in pediatric rheumatologic diseases, where it has demonstrated a favorable safety profile. We thoroughly discussed all possible treatment options and associated risks with the patient’s parents and finally obtained consent for treatment with rituximab. In our patient, treatment with rituximab resulted in good disease control and an excellent outcome. During the follow-up period, he experienced no further relapses or radiological progression of the disease and no serious adverse events.

We believe that our experience is valuable and will contribute to a better understanding of disease progression in patients with very early-onset MS and to the clinical evaluation, management, and treatment of these patients. Further prospective studies are needed to decide on the best therapeutic approach in such young children.

In conclusion, early recognition and treatment are beneficial for favorable outcome in very early onset POMS patients. Further observational and prospective studies in this patient population are warranted to provide new recommendations for the best treatment options.

## Data availability statement

The original contributions presented in the study are included in the article/supplementary material, further inquiries can be directed to the corresponding author.

## Ethics statement

The studies involving humans were approved by National Medical Ethics Committee of the Republic of Slovenia. The studies were conducted in accordance with the local legislation and institutional requirements. Written informed consent for participation was not required from the participants or the participants’ legal guardians/next of kin in accordance with the national legislation and institutional requirements. Written informed consent was obtained from the minor(s)’ legal guardian/next of kin for the publication of any potentially identifiable images or data included in this article.

## Author contributions

UA: Writing – original draft, Conceptualization, Data curation, Investigation, Methodology. TVV: Writing – original draft, Data curation, Investigation, Visualization. DO: Writing – review & editing, Resources, Supervision. MPB: Writing – review & editing, Conceptualization, Supervision. NB: Writing – original draft, Writing – review & editing, Conceptualization, Data curation, Formal analysis, Funding acquisition, Investigation, Methodology, Supervision, Validation.
